# Detection and identification of cis-regulatory elements using change-point and classification algorithms

**DOI:** 10.1186/s12864-021-08190-0

**Published:** 2022-01-25

**Authors:** Dominic Maderazo, Jennifer A. Flegg, Manjula Algama, Mirana Ramialison, Jonathan Keith

**Affiliations:** 1 School of Mathematics and Statistics, The University of Melbourne, Melbourne, 3010 VIC Australia; 2 School of Mathematics, Monash University, Melbourne, 3800 VIC Australia; 3 Australian Regenerative Medicine Institute, Monash University, Melbourne, 3800 VIC Australia

**Keywords:** Conserved non-coding sequences, Putative functional elements, Genome segmentation, Bayesian modelling

## Abstract

**Background:**

Transcriptional regulation is primarily mediated by the binding of factors to non-coding regions in DNA. Identification of these binding regions enhances understanding of tissue formation and potentially facilitates the development of gene therapies. However, successful identification of binding regions is made difficult by the lack of a universal biological code for their characterisation.

**Results:**

We extend an alignment-based method, changept, and identify clusters of biological significance, through ontology and de novo motif analysis. Further, we apply a Bayesian method to estimate and combine binary classifiers on the clusters we identify to produce a better performing composite.

**Conclusions:**

The analysis we describe provides a computational method for identification of conserved binding sites in the human genome and facilitates an alternative interrogation of combinations of existing data sets with alignment data.

**Supplementary Information:**

The online version contains supplementary material available at (10.1186/s12864-021-08190-0).

## Background

At the transcriptional level, gene expression is largely mediated through the binding of proteins to non-coding regions in deoxyriboucleic acid (DNA). This is achieved by directly stabilising or blocking the binding of riboucleic acid (RNA) polymerase, or by interacting with other proteins and co-factors capable of influencing transcription [1]. These transcription factors (TFs) binding to *cis*-regulatory elements (CREs) are responsible for initiating, modulating and terminating transcription.

CREs are often in neighbouring non-coding regions of target genes and can consist of multiple sites at which TFs can bind. Such binding sites are typically short (6-30 base pairs) degenerate sequences and are often located in promoter regions within a few kilobases (kb) either side of the transcription start sites (TSSs) of target genes [2]. However, the regulatory landscape is not limited to the immediate vicinity of a gene. Some TFs depart from the common pattern of binding near TSSs; for example, in mouse embryonic stem cells, the TF Smad1 has more than 86% of its binding sites at a distance greater than 5 kb from the TSS of any gene, and in fact its binding sites are depleted near TSSs [3–5]. Classes of more distal binding regions known as *cis*-regulatory modules (CRMs) comprised of different CREs have also been identified to play important roles in transcriptional regulation [6]. Projects involving the mapping of the human regulatory genome have revealed that TFBSs are more widely distributed, even being located hundreds of kb away from the nearest gene [7–10]. Thus, the location of TFs in relation to the regulatory domain of their target genes is likely variable per gene.

Varying genomic factors such as sequence specificity, chromatin accessibility, protein-protein interactions, epigenetic modifications to the DNA and histone structure contribute to TF binding to their target sites [11–13]. The reorganisation of these chromatin structures allows for variability in transcriptional states and subsequent expression patterns [14, 15]. Factors such as these can vary through the life-cycle of a cell, allowing for the expression of different profiles required of a cell to proceed down a particular developmental pathway or to carry out specific functions.

We proceed by using a Bayesian segmentation-classification algorithm changept rooted in comparative genomics. This algorithm has been previously successful in the identification of conserved non-coding regions [16]. In summary, this method delineates an alignment and classifies segments according to their structure within the alignment. In this paper, we extend previous methods to a genomic scale by clustering segments obtained from individual chromosomes. We then investigate these clusters for putative functional elements (PFEs), contained within the identified segments, through ontology analysis and enrichment for binding motifs. We also use a method for comparing and combining binary classifiers that assess the functional relevance of genomic regions based on the presence of certain genetic markers. The resulting combined classifier has improved sensitivity and specificity relative to its constituent classifiers. We find that combinations that use markers based on chromatin immuno-precipitation followed by sequencing (ChIP-Seq) and DNase Hypersensitivity (DHS) outperform those that do not, possibly indicating the presence of enhancers within these clusters. Lastly, we find that changept is able to identify clusters that are enriched in different ontology terms and statistically significant motifs, which together hint at the shared biological function of the segments withing a cluster.

## Results

The downloaded alignments were partitioned by zebrafish chromosome to give 25 smaller alignments for encoding. Alignments of zebrafish, mouse and human sequences were obtained by removing the other five species from the 8-way alignment and discarding any alignment blocks that did not contain all three species of interest, resulting in 3-way alignments that were encoded.

### Clustering

The silhouette method for model selection identified 16 clusters by applying *k*-means clustering on the segment class character frequencies. Approximately 89% of the variation between the segment classes was captured by the first two principal components with the largest loadings attributed to the characters v and a, respectively. This is of note since these are the characters corresponding to completely conserved bases within the alignment. These clusters are available in genetic (hg19) coordinates in .bed format via the cluster beds folder in figshare link in the data availability section.

We also identified 422 segments overlapping with known conserved non-coding regions presented by Babarinde and Saitou [17]. The majority of these segments were located in clusters 4 (248), 7 (76) and 12 (69): clusters with high conservation levels (see Table 1). However, clusters with lower conservation levels, such as clusters 13 and 14, also contained segments overlapping with known conserved non-coding regions.

**Table 1:** Summary statistics for segments identified in clusters

Cluster	*M*_*c*_	*M*_*s*_	$M_{s}^{*}$	*η*
1	21	1437	641	0.494
2	28	2804	2646	0.321
3	27	8439	3973	0.722
4	24	1714	1263	0.745
5	29	1590	1144	0.388
6	10	102	88	0.320
7	14	725	562	0.556
8	18	1361	610	0.572
9	25	819	655	0.246
10	14	2340	1127	0.594
11	28	2297	1181	0.522
12	28	9083	4219	0.695
13	24	506	460	0.261
14	22	702	643	0.342
15	28	2329	1341	0.422
16	22	103	94	0.380

Here *M*_*c*_ is the number of segment classes, *M*_*s*_ is the number of segments, $M_{s}^{*}$ is the number of segments not overlapping with UCSC exons, *η* is the average conservation level of non-exonic segments

### Estimating the performance of individual classifiers

**Fig. 1:**
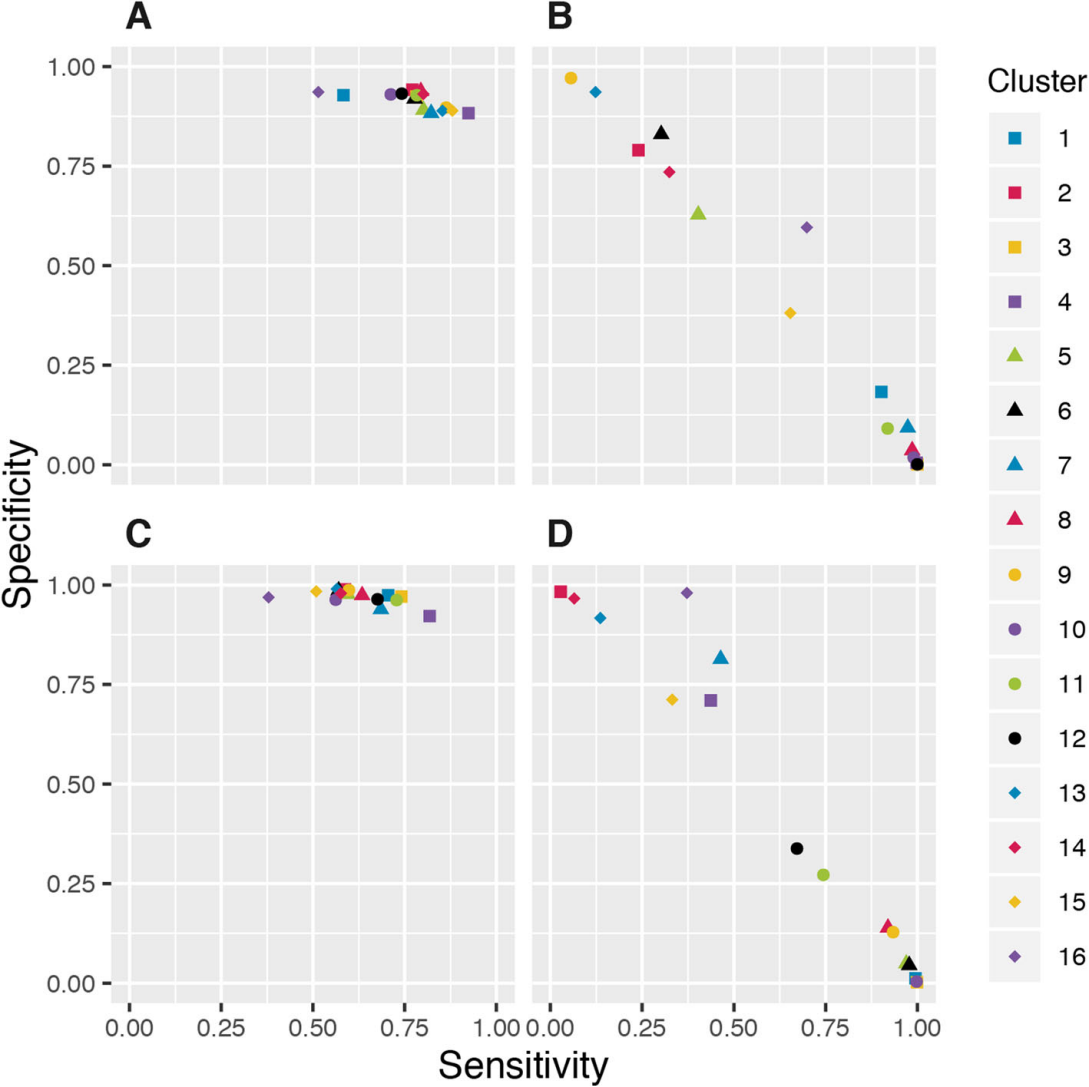
Sensitivity and specificity estimates. The estimated performance of each of the four binary classifiers under the model for each cluster. (A) ChIP-Seq. (B) Conservation level. (C) Dnase-Seq. (D) GC content

Performance estimates for the four binary classifiers are visualised in Fig. 1 and also available in the figshre project under the Classifier estimates folder. We observe that, as individual classifiers for a segment containing a PFE, DHS and ChIP-Seq outperform GC content and conservation level using the thresholds that we have set. This can be seen in the tighter grouping and more consistent specificity in the scatter plots for DHS and ChIP-Seq. Conservation and GC content, considered as individual binary classifiers, performed inconsistently across clusters. In the case of both of these, a high sensitivity was usually accompanied by low specificity and *vice versa*. The varied performance across the clusters could mean that neither conservation nor GC content would be suitable for use as the only classifier in an analysis seeking to identify regions containing PFEs, in contrast to the more consistent performance of DHS and ChIP-Seq, which are features typically used in functional element discovery. A reciever operating characteristic curve containing the aread under the curve values for the GC classifier used on the clusters presented in the discussion section is available in Additional file 1.

### Identifying the optimal combination of classifiers

**Fig. 2:**
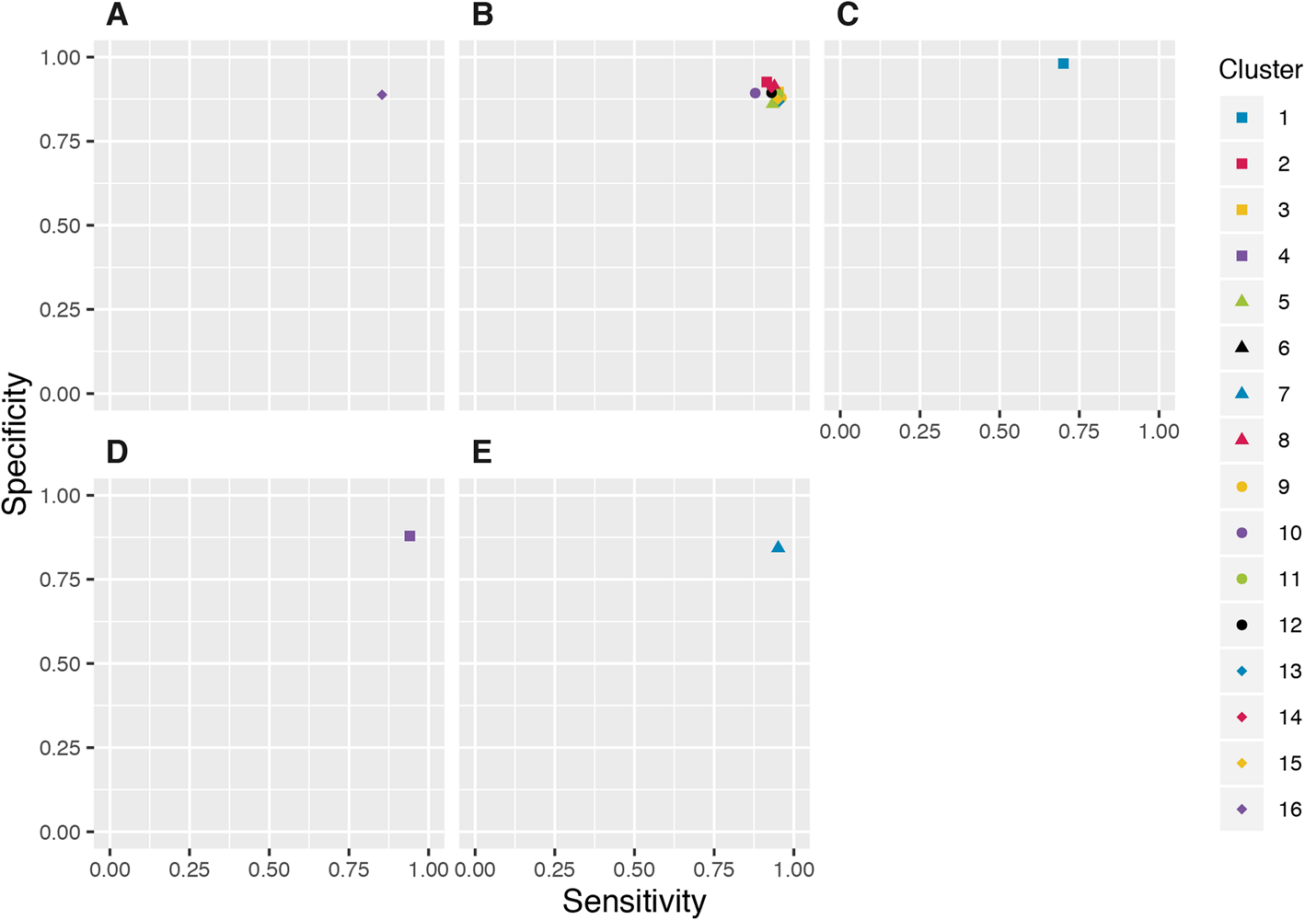
Sensitivity and specificity estimates for combinations. Clusters identified different combinations as optimal under our selection criteria. (A) Union of DNase-Seq, ChIP-Seq and GC content. (B) Union of Dnase-Seq and ChIP-Seq. (C) DNase-Seq. (D) ChIP-Seq (E) At least two of DNase-Seq, ChIP-Seq or conservation level

For each cluster, we also investigated ways of creating a composite classifier with improved performance relative to individual classifiers (for the purpose of identifying segments that contained conserved non-coding functional elements). Through this process, we identified five different combinations of classifiers deemed optimal in at least one cluster according to our selection criteria. The performance of these combinations is visualised in Fig. 2 and are also available under the Classifier combinations directory of the figshare project. We observe that for the majority of clusters, the top ranking combination identified was simply the union of DHS and ChIP-Seq. This particular combination is consistent with studies using a combination of these two types of data to characterise functional regulatory elements and identify novel putative elements [18, 19]. For a segment to be classified with a ‘1’ using this composite classifier, it would have to have a ‘1’ classification for at least one of the individual DHS or ChIP-Seq classifiers, regardless of classifications obtained using the GC content or conservation.

Classifiers that were composed of positive classifications for either ChIP-Seq or DHS tended to perform better than those that did not include them. From a biological perspective, these two data types come from experimental and verified data collection. On the other hand, the GC content and conservation classifiers as we have defined them are synthesised from a sequence alignment. However, there were two clusters that identified optimal combinations which included the GC and conservation classifiers.

In clusters 7 and 16, the optimal combined classifier included conservation and GC content, respectively. The identified combination in cluster 16 included the union of having above threshold GC content with the union of DHS and ChIP-Seq as the best performing. This classifier would classify a segment as a putative functional element if it had a positive result in at least one of DHS, ChIP-Seq or GC content. The composite classifier identified in cluster 7 selects regions that have a positive result in at least two of DHS, ChIP-Seq or above threshold conservation.

In most cases, the composite classifier identified for each of the clusters results in an increase in both the sensitivity and specificity compared to each of the individual constituent classifiers. However, we do observe that for some clusters, the identified optimal combination of classifiers may have reduced sensitivity or specificity when compared to an individual classifier. For example, in clusters 7, 8 and 12, conservation as an individual classifier had very high sensitivity but low specificity. Theoretically, a classifier with a sensitivity of 1 is able to correctly identify a true positive all the time. However when this is combined with a low specificity, as in the case with the conservation classifier for these clusters, this classifier fails at identifying any true negatives. In these cases, the composite classifiers produce a drop in sensitivity but gains in specificity. This translates to a drop in the rate of true positives but an increase in true negatives, thus making the identified combination a better performing classifier overall.

### Ontology and motif analysis

We next investigated whether the clusters of regulatory elements identified by changept relate to known biological functional groups. For this, human genomic coordinates of the segments in each cluster were analysed for enrichment of ontology terms using the genomic regions enrichment of annotations tool (GREAT) platform with standard association rules [20] (Table 2). The full outputs for each cluster can be found in the figshare project. In cases where clusters had association with ontology terms presented by GREAT, the most commonly enriched ontology type in the clusters were biological process terms (173), which describe genetically programmed objectives that organisms are seeking to achieve. This was followed by molecular function terms (9) describing tasks or activities of gene products rather than components or end products, and finally cellular component (5), which refers to the structures and bio-molecules that make up a cell.

**Table 2:** The number of gene ontology (GO) terms and mouse phenotypes with knock out (KO) identified for each cluster

	Cluster															
	1	2	3	4	5	6	7	8	9	10	11	12	13	14	15	16
GO Biological Process	10	2	17	69	4	-	12	-	3	-	22	27	-	2	5	-
GO Cellular Component	-	-	-	-	1	-	2	-	-	1	-	-	-	1	-	-
GO Molecular Function	-	-	1	4	-	-	-	-	1	1	-	-	-	1	1	-
Mouse Phenotype Single KO	-	2	16	136	-	-	6	-	-	2	3	13	-	3	-	-
Motifs identified	1	2	1	2	1	-	-	-	2	-	-	1	1	1	-	-

Examination of the different ontology terms and associated genes present in each cluster revealed that there was variation across clusters, being aligned with different biological systems or processes. It is important to note that not all clusters showed an enrichment in specific annotation categories. In our analysis, we find that 12 out of the 16 clusters identified showed enrichment for ontology terms, suggesting that there is some shared function of the segment regions in each cluster. In cases where there was no enrichment for ontology terms, this may reflect the potential for changept to detect clusters of shared, but as yet unknown, biological function.

To further investigate whether the clusters of segments that our method had created were of biological significance, de novo motif analysts was performed with Trawler in order to identify shared DNA binding motifs within each segment. The analysis undertaken with Trawler revealed that some of these clusters are also enriched for motifs. We find that 9 out of 16 clusters show an enrichment for statistically significant motifs, with three of the 9 showing enrichment for more than one motif. The presence of these motifs may suggest the regions in a cluster co-regulate multiple genes. Alongside the graphs described above are motifs of TFBS that have been identified by Trawler to be present among the segments of that cluster accompanied by a sample of some of the genes that may be associated with those particular motifs. Just as with the ontology terms, all the motifs identified for each cluster are available in the relevant folder in the figshare project.

## Discussion

In the following section, we discuss some possible biological functions for several of the clusters identified in the analysis. Given that all the terms within a cluster are statistically significant for the 0.05 false discovery rate threshold, a graph of the most frequent words for each ontology type is created to summarise the terms that are observed in each cluster for the tissue, system or process terms associated with a cluster. Together with the motifs identified through de novo motif analysis with Trawler, we demonstrate that the clusters that are predicted through this method are enriched for biological pathways which share common motifs. The motifs are also presented for the relevant clusters, along with predicted TFBS, as we further investigate the biological significance of these enrichments.

### Known regulatory element features are retrievable by changept

It is well-known that developmental genes, in particular transcription factors and genes associated with neuronal functions, are regulated by highly conserved enhancers [21, 22]. In accordance with this, we have identified 2 clusters (Clusters 4, 15) in which segments identified via changept were found to be associated with genes with DNA-binding and neuronal function as identified by GREAT.

**Fig. 3:**
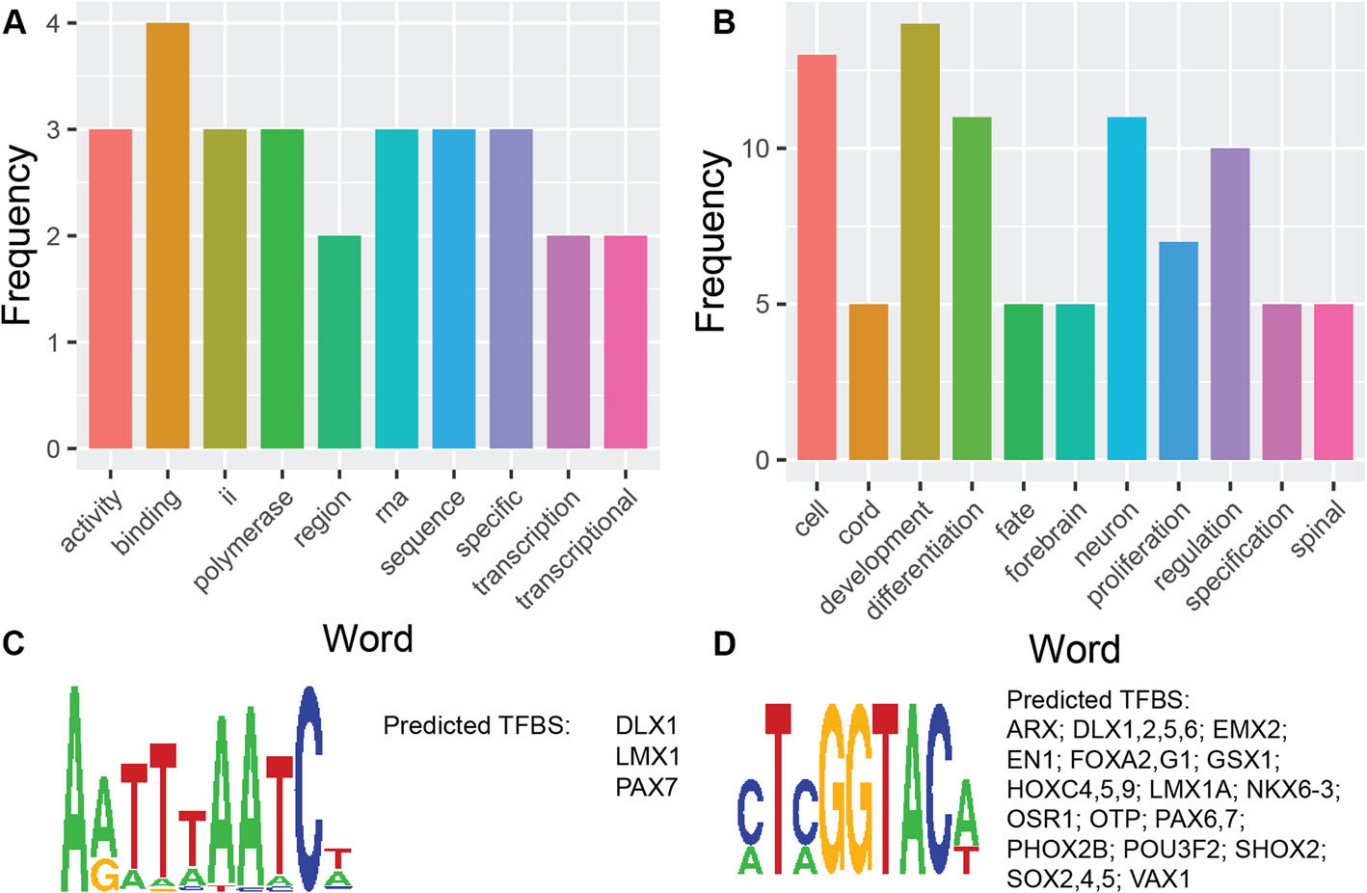
Cluster 4 ontology word frequencies and motifs. Results from GREAT displayed as the frequency of specific words appearing in different types of ontology terms. Motifs presented are obtained from Trawler. (A) Molecular function for cluster 4. (B) Biological processes for cluster 4. (C) Motif 1 and predicted TFBS (D) Motif 2 and predicted TFBS

The gene ontology (GO) terms for DNA-binding transcription repressor activity, RNA polymerase II-specific (GO:0001227) as well as high mobility group box domain binding (GO:0071837) molecular function terms were enriched in cluster 4 (Fig. 3A). DNA binding (GO:0003677) was also enriched within cluster 15. A number of segments present in cluster 15 were also shown to be in the regulatory domains for a number of genes in the zinc finger (ZNF) TF family. It has been established that these elements are commonly associated with protein binding and assisting in developmental regulation [23, 24]. Newer evidence suggests that ZNFs display flexible binding characteristics, also binding to RNA, lipids and post translational modifications and playing broader biological roles such as protecting genome integrity and telomere maintenance [25].

Regions in both clusters fell in to the regulatory domains for several genes in the Pit, Oct, and Unc (POU) family of transcription factors; *POU3F2*, *POU3F3*, *POU4F2* and *POU5F1B*. This particular family of genes plays various regulatory roles in development, metabolism and immunity and this range is observable across the specific examples we encounter. Cluster 4 was enriched for numerous biological process terms associated with neuronal development such as regulation of neural retina development and cell proliferation in forebrain (GO:0061074, GO:0021846) (Fig. 3B) These ontology terms show associations with POU3F3 and POU4F2, known for regulatory roles in neuronal and other tissues. In the case of POU3F3, it has been found to be a regulatory component for cortical neuron migration in the development of the brain [26]. While POU4F2 has been linked to neural retina development, it has also been linked to adaptive hypertrophic responses in hearts [27]. Further illustrating the range of systems that the POU family play roles in we have POU5F1B, associated with terms in cluster 15, which has been found to have roles in cancer cells both in vitro and tumours in vivo [28, 29].

de novo motif discovery performed by Trawler on sequences in Cluster 4 revealed two statistically significantly enriched motifs (Fig. 3C-D) Putative transcription factors known to be associated with these motifs include members of the Homeobox and the POU family of transcription factors (Fig. 3C-D). Interestingly, Cluster 4 contains segments that were associated by GREAT to genes belonging to these specific transcription factor families (e.g. paired box 7 (PAX7), POU3F2) This implies that these transcription factors regulate their own expression by binding to their own regulatory region. This is consistent with current knowledge about these transcription factor families that are known to self regulate [30].

Altogether, this demonstrates that changept is able to retrieve clusters that represent known biological groups of genes such as developmental genes which are co-regulated by common TFBSs under high evolutionary constraint.

### Change-point identifies synexpression groups

Our analysis further identified examples of clusters showing enrichment in only one type of gene ontology; the GO biological processes. The absence of enrichment in the cellular component GO category suggests that genes belonging to these clusters do not originate from a particular location in the cell. Similarly, the absence of enrichment in the molecular function GO category suggests that genes belonging to this cluster encode for different classes of proteins. Altogether, we propose that these clusters represent synexpression groups. Indeed, synexpression groups are characterised by a group of genes that are not molecularly related but act synergetically to ensure that a specific functional program is achieved [31] by the shared regulation of the genes belonging to this group [32].

**Fig. 4:**
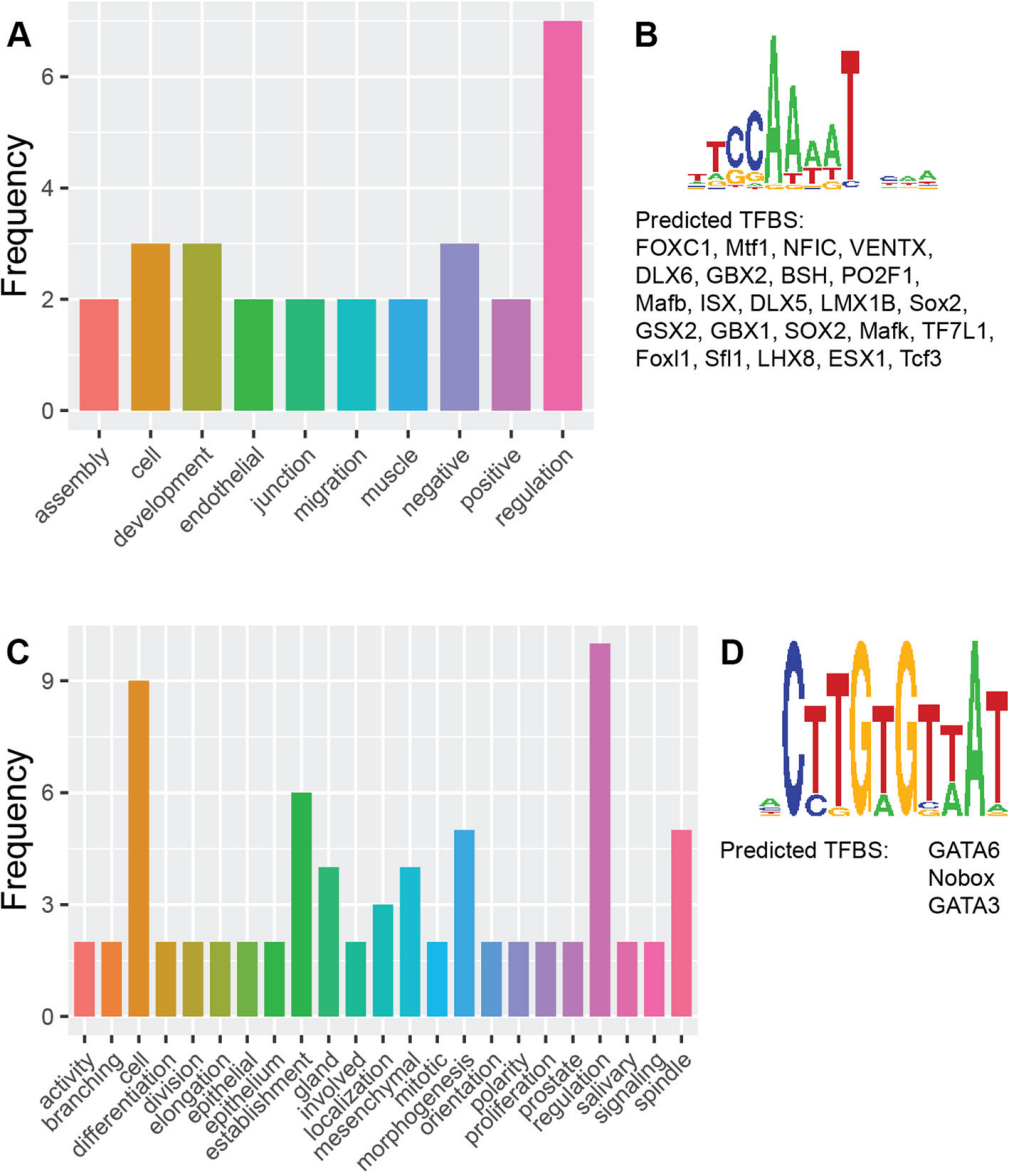
Ontology word frequencies and motifs. Results from GREAT displayed as the frequency of words appearing in different types of ontology terms. Motifs presented are obtained from Trawler. (A) Biological processes for cluster 1. (B) Motif for cluster 1. (C) Biological processes for cluster 12 (D) Motif for cluster 12

For instance, the most significantly enriched terms in Cluster 1 are for the development of muscle organ and structure (GO:0007517, GO:0061061) This is followed by various terms, among which are the regulation of endothelial cells (GO:0010594, GO:0010595) An overview of terms associated with this cluster can be seen in Fig. 4A. Genes associated with these terms, such as *Wnt3a*, have been shown to increase fibrosis in muscle cells [33]. Other genes, for example *Sox6*, have been linked to structural changes in skeletal-muscular cells of mice [34] or delayed muscle regeneration in zebrafish [35]. Further, de novo motif discovery with Trawler identified a motif which was a known binding site for a range of TFs known to be developmental regulators of different muscle tissues (Fig. 4B). The TFs identified to bind to this motif include forkhead box C1 (FOXC1), which plays critical roles in early cardiogenesis [36] and LIM homebox 1B which regulates the development of ocular muscles [37]. Altogether, these data support that Cluster 1 represents a synexpression group dedicated for muscle function. The enriched biological process GO terms in Cluster 12 relate to cell cycle regulation. There are terms for regulation of cell division (GO:0051302), the establishment and maintenance of cell polarity (GO:0030010, GO:0007163) as well as for the establishment and localisation of spindle apparatus (GO:0000132, GO:0040001, GO:0051294, GO:0051293) (Fig. 4C) *CDK5RAP2* was found to be associated with a number of the terms. The protein encoded by this gene is present during mitosis, localised in the spindle poles and it has been found that this gene is essential for cell proliferation in the cerebral cortex [38]. Mutations in this gene have also been clinically observed to induce micro- cephaly and sensorineural hearing loss in humans [39, 40]. Interestingly, the mouse phenotype terms showed links to various types of abnormal physiology, particularly in the brain, adding support for association of this cluster with brain structures. De novo motif discovery with Trawler also revealed an enriched motif that is a known binding target for GATA3 and GATA6 (Fig. 4D): TFs implicated in the regulation of the cellular cycle. Irregular levels of these TFs play a role in different cancers [41, 42]. We also identify several segments that fall within the regulatory regions for *cyclin D1*, which is a known target of GATA3 in tumour cells [43]. Altogether, these findings suggest Cluster 12 is a synexpression group dedicated for the regulation of the cell cycle in brain structures.

### Change-point identifies co-regulated genes belonging to distinct subcellular units

**Fig. 5:**
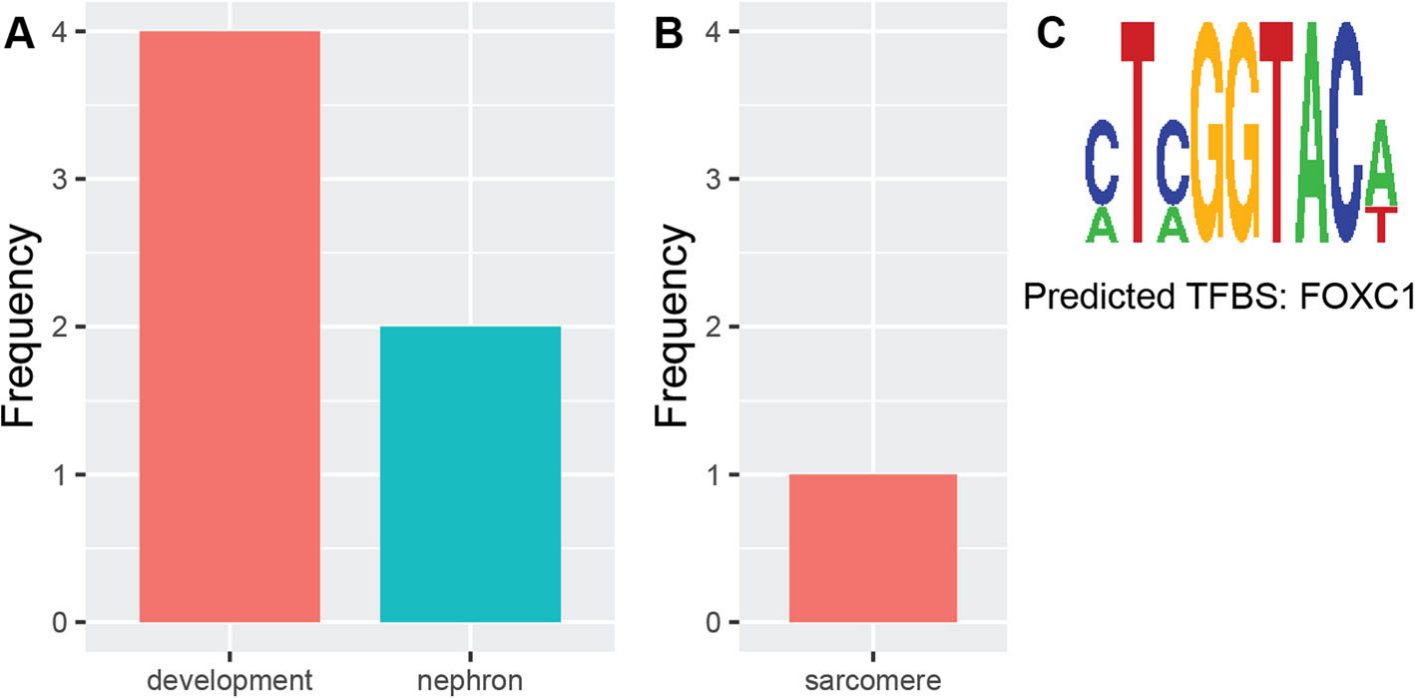
Ontology word frequencies and motifs for cluster 5. Results from GREAT displayed as the frequency of words appearing in different types of ontology terms. Motifs presented are obtained from Trawler. (A) Biological processes. (B) Cellular component. (C) Motif and predicted TFBS

In this analysis, the cellular component ontology term was present in the least number of clusters, however some clusters do show enrichment in particular cellular substructures, suggesting that genes implicated in the formation of these cellular substructures share common regulation. For instance, the sarcomere is a cellular component usually associated with muscle tissue, giving these cells a striated formation, particularly in the heart; terms relevant to the sarcomere were identified in conjunction with terms for biological processes (Fig. 5A). Defects in sarcomeric proteins are the primary attributes of hypertrophic cardiomyopathy. However, modern analysis is revealing that the heterogeneity in hypertrophic cardiomyopathy may be attributed to defects in secondary genes like *cysteine and glycine rich protein 3 (CSRP3)* [44, 45]. Our analysis produced segments within Cluster 5 that were associated with sarcomeric genes (including *CSRP3*), leading to the enrichment of the sarcomere term (GO:0030017) for cellular components (Fig. 5B) In accordance with this function, the motif identified in this cluster was also a known motif bound by FOXC1, which is a crucial transcription for heart development and is known to be essential for cardiomyocyte formation (Fig. 5C) Cluster 7 also presented cellular components for growth cone (GO:0030426) and sites of polarised growth (GO:00300427) These terms are closely linked to neuronal cells’ ability to grow in a particular direction, in search of axonal targets. Segments in this cluster showed association to the regulatory domains of *fibronectin leucine rich transmembrane (FLRT)* and *ELKS/RAB6-Interacting/CAST family member 2 (ERC2)*. Both these genes have been observed to affect aspects of neuronal features within model organisms. In particular, there have been links to decreased synaptic activity in FLRT knock out mice as well as decreased neurotransmistter release and synaptic transmission in down regulated ERC2 mice [46, 47].

Altogether, this data indicates that changept is able to identify clusters of genes sharing a common cellular location. These might represent subsets of synexpression groups as evidenced by the enriched motif in Cluster 5.

### Validity of results

Trawler identifies statistically significant motifs present within the clusters or regions that we determine through the changept analysis and additionally matches PWMs of these motifs against databases of TFBS, such as JASPAR. These databases make use of a variety of data types, sourced from multiple protocols such as SELEX and ChIP-Seq. Hence, the motifs that are presented by Trawler, and present within our clusters, can theoretically be recognized by the suggested TFs. Additionally, approximately 20% of the regions identified through this analysis overlap when compared to the phylogenically conserved TFBS track on UCSC. However, the claim that novel putative functional elements can be identified via changept analysis requires additional validation.

To validate the regions identified by this analysis, we compared the regions that contained a predicted TFBS from Trawler against the Encode regulatory TFBS track available on UCSC. These tracks represent known binding locations of TFs. The comparison is carried out by comparing regions harbouring predicted TFBSs that are present in the cluster under consideration with the UCSC TFBS tracks. This then allows us to identify overlaps with the Encode track and determine a list of TFs that are known to bind within the segments in the clusters. We identify that there is an overlap in a subset of the predicted TFs from Trawler and the TFs from the Encode track. Out of the 4 clusters investigated which had predicted TFBS, we found an occurrence of the predicted TFBS that was overlapping with an existing TFBS from the UCSC track in 3 of these clusters. Hence, we assert that the regions identified by changept in this analysis must have biological significance in the form of regulatory potential.

## Conclusion

We have presented a comparative, alignment based approach to identify clusters of conserved putative functional elements using the program changept. This method was demonstrated in this paper using a 3-way zebrafish-referenced alignment, but similar analysis can be carried out with the substitution of any 3-way alignment. This method can be performed on 2-way alignments and extended to 4-way alignments, given a suitable choice of alignment encoding. Clusters that were identified in this analysis through their representative structure by changept differ in biological significance and function, and are associated with different tissues or processes elucidated through gene ontology analysis.

Novel functional element discovery can be a costly and time consuming process partially due to the nature of investment required in assets such as reagents and cell lines as well as experimental design. While large volumes of ChIP-Seq data continue to be generated, new functional elements being discovered rely on some level of apriori knowledge for the design of the experiment to find the appropriate targets. The advantage of this approach is that it does not require any apriori assumptions, although such assumptions can be incorporated if desired. Other than some knowledge based on a multiple sequence alignment, our approach can identify significant regions of potential function and this may open the door to the discovery of functional elements that may not otherwise be found using traditional means.

The method as we describe it is illustrative of the way that such analysis of this nature can be performed. However, it provides sufficient scope to tailor the analysis being performed to more targeted classes of cis-regulatory elements. This can be done, for example, by using a different alignment where promoters are more prominently conserved and using classifiers that are more strongly correlated to the type of element of interest. Alternative clustering approaches could be incorporated to discern the functional segments identified for specific tissues or processes according to the relevant metric.

Further, we have demonstrated a method for estimating the sensitivity and specificity of binary classifiers for putative functional element discovery. We have used these estimates to identify composite binary classifiers with improved performance over their constituent classifiers. We observe that classifiers based on DHS and ChIP-Seq outperform those based on GC content and conservation level, as we have defined them, as stand-alone classifiers. Analysis of all combinations of binary classifiers revealed that the best combination available was typically the union of DHS and ChIP-Seq and that the composite classifier formed from the union of these also performed well.

Our analysis suggests that regions enriched with both DHS and ChIP-Seq are potential candidates for TFBS. This is consistent with the literature identifying these two features being common among regulatory elements such as promoters and enhancers [48, 49]. However, this combination of enrichment may still result in mis-identification of non-coding functional elements as they may indeed be binding sites but for proteins that do not play a regulatory role in gene expression. Optimal combinations of classifiers identified in this study tended to exclude classifiers based on GC content and conservation level, suggesting these classifiers are not contributing useful information in this context. Currently, the number of classifiers for which the optimal combination can be identified is limited to four or five by computational constraints, but in current work we are attempting to increase this number.

## Methods

### Alignment of data

We extend the analysis from the previous work of Algama [16] and proceed by using the same dataset. A zebrafish referenced multiz 8-way alignment in.maf format was obtained from the University California Santa Cruz (UCSC) genome browser at http://hgdownload-test.cse.ucsc.edu/goldenPath/danRer7/multiz8way/ and split into 25 data sets corresponding to zebrafish chromosomes, where alignment blocks that overlapped with RefSeq genes were removed. These were then uploaded to the galaxy platform (usegalaxy.org) to extract 3-way alignments containing zebrafish (danRer7), mouse (mm9) and human (hg19) sequences and to ensure that all alignment blocks contained no less than 7 columns [50, 51]. From the point of view of the human genome, the regions under consideration are the subset of sequences found in intronic, intergenic and promoter regions that are mappable to the other organisms within the alignment.

### Alignment encoding

The minimum input required for changept is a binary sequence. However, for this application a, 3-way alignment was converted into a single sequence using a 32-character code (see below). Any alignment columns containing indel characters, represented by a ‘-’, were encoded with special characters and ignored within the analysis; characters on either side of an indel were considered to be adjacent. The encoding is summarised as,


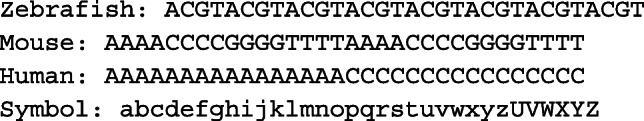


Alignment columns with a G or T in the human sequence were complemented and then encoded using the same characters. Non-contiguous alignment blocks were separated using the # symbol; these are considered as fixed change-points by the model. This encoding was chosen as it encapsulates information about the three sequences, such as alignment structure, guanine-cytosine (GC) content and conservation.

### Change-point analysis

#### Overview

The encoded sequences generated from each of the alignments corresponding to zebrafish chromosomes were independently run through program changept to perform segmentations. A full description of the model can be found in previous papers [52–54].

Briefly, changept is a Bayesian segmentation-classification algorithm. The input for the algorithm is a single sequence. For our purposes, we used the 32-character code described above. The algorithm then iteratively produces a segmentation, delineating the input sequence *S* by finding the change-point positions, represented by a vector *C*. Segments are then allocated into one of *T* segment classes, with the vector *g*, where *g*_*i*_∈{0,…,*T*−1}, used to assign membership of each segment to a class. The character frequency of a segments in a class is modelled by a multinomial distribution with parameter *θ*=(*θ*_*a*_,…,*θ*_*z*_,*θ*_*U*_,…,*θ*_*Z*_) which is drawn from one of *T* dirichlet distributions with parameter vector *α*. The number of segment classes in a model is unknown a priori and is determined through model selection.

**Fig. 6:**
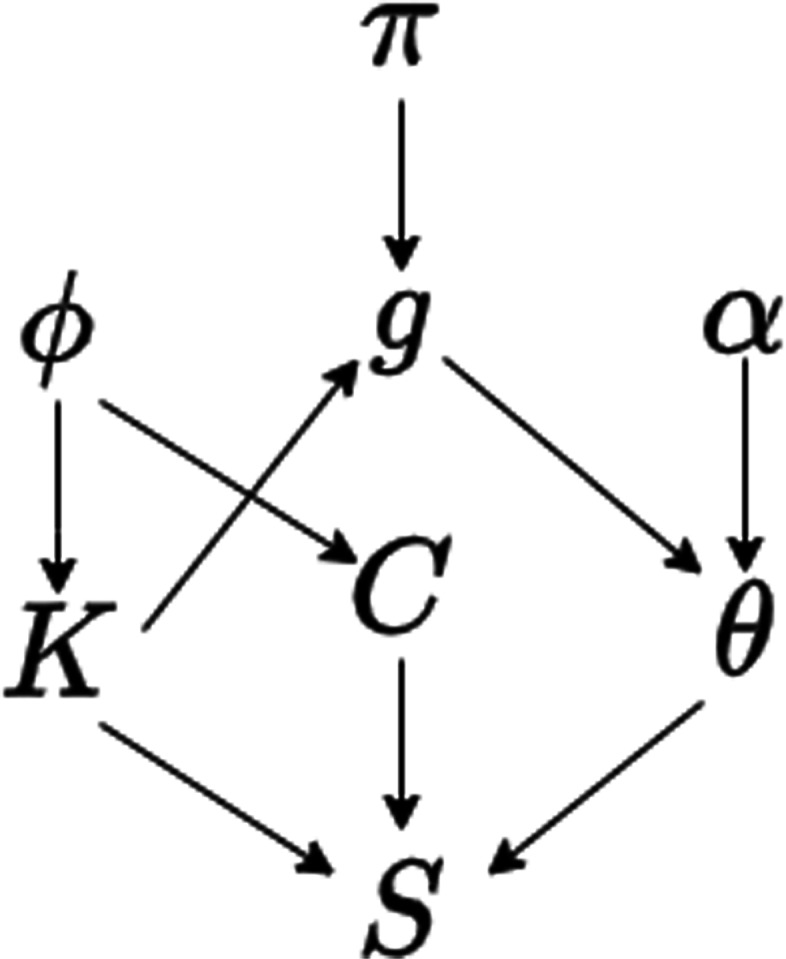
Conditional dependence diagram. The parameters for the changept model. Parameters at the head of an arrow are conditionally dependent on the parameters at the tail. The parameter *ϕ* governs the probability of a change point at a position and the parameter *π* is a probability vector that governs the segment allocation to a class membership, also known as the mixture proportion

The Generalized Gibbs Sampler [52] was used to generate samples from the varying-dimensional space, since parameters such as the number of change points *K* are also unknown. Upon completion, the algorithm produces posterior probability estimates of the locations of segment boundaries and posterior probabilities of each position in the sequence being assigned to a segment class. These probabilities were obtained using the program readcp (part of the changept suite) and an overview of the conditional relationships between the parameters of the model can found in Fig. 6.

#### Tuning and model selection

Tuning and model selection was required in order to determine the number of segment classes *T* in the model. Independent runs with different values of *T* were performed and used to determine the optimal number of classes for each alignment, using approximations to the Aikake and bayesian information criterions, and a variant of the deviance information criterion utilizing variance, all calculated for post burn-in samples. Discussed in Oldmeadow and Keith [55], the robustness of this criteria was demonstrated in Bayesian segmentation classification algorithms in multiple circumstances. Models with the lowest values of these information criteria are generally preferred, as they represent a compromise between model fit and the effective number of parameters. In combination with this method, the model selection procedure outlined previously by Algama et al. [56] was used. In this approach, a model containing segment classes with very low mixture proportions (<0.5*%*) was considered over-fitted and discarded in favour of models with a smaller number of classes.

Due to the probabilistic nature of the algorithm and its reliance on posterior distribution samples obtained via Markov chain Monte Carlo, it was necessary to establish whether convergence to the limiting distribution had occurred. This was done by inspecting trace plots of the log-likelihood for each of the iterations as well as for parameter estimates to identify the *burn-in* period, which was characterised by an upward trend in the log-likelihood. For the following analysis, only samples taken after burn-in were used. The steps of this workflow are summarised in Appendix Fig. 1 (located in Appendix A) in a flow diagram. However, a complete description of the changept and readcp programs can be found in [57].

#### Segment retrieval

For each dataset, the readcp output was used to identify segments for further analysis after segmentation with changept. Only segments satisfying the following criteria were considered for further analysis:

- minimum segment length - 6 nt
- minimum profile value - 75%
- maximum gap size in a segment - 3 nt
- maximum proportion of gaps in a segment - 50%

As we are seeking PFEs, the first criterion reflects a minimum size for an individual site of a TFBS [58, 59]. The other criteria are related to technical considerations that arise when using changept. Segment assignment to classes is probabilistic, and requiring segments to have a high probability of belonging to a specific segment class is thus recommended to avoid false positive allocations; we therefore prescribed a minimum profile value of 75%. changept considers characters on either side of a gap to be adjacent, so a segment containing long gaps or a large proportion of gaps may not be indicative of real genomic structure. These criteria were a slight modification of those used previously by Algama et al. [16], where changpt was used to identify conserved non-coding regions. We have opted for a more relaxed value of minimum segment length and profile value to allow for more potential candidates for conserved non-coding regions and a more aggressive criteria for the gap size to compensate.

### Clustering

Within a data set, segments were classed and characterised by the frequencies of characters obtained from the encoded representation of the segment. Up to this point in the analyses, the data sets were treated independently, but from here the segment classes across the data sets are clustered to identify correspondence between data sets and pool clusters from different segmentations. The character frequencies for each of the different segment classes are all collected into a single.csv file in the Character frequencies folder of this figshare project.

The segment classes were clustered by assigning the proportion of a character frequency as a dimension in Euclidian space and then using a standard *k*-means clustering algorithm. Model selection was done using the silhouette method [60]. Segment classes identified to be in the same clusters had their representative segments grouped together and any segments found to overlap with UCSC exons were excluded for the remainder of the analysis.

### Evaluating and combining classifiers

#### Classifier data

In the 32-character representation used to encode the 3-way alignments, characters a and v represent the bases across all three species are conserved in an alignment column, while the characters q through to Z represent the GC content in the human species. The conservation and GC content of a segment is defined to be the proportion of characters in the encoded sequence corresponding to the feature of interest and classification is carried out by setting a threshold for these proportion. Although the GC content and conservation can be directly computed by the representation of the sequence using the encoded alphabet, the current implementation of our algorithm requires binary input. When classifying for GC content, a segment is positively classified if its encoded representation is comprised of greater than 40% of the characters q through Z; any segments failing this criteria are classified negatively. In a similar fashion, a segment is positively classifed for conservation if greater than 40% of the encoded representation are the characters a or v and negatively classified otherwise.

DNase-Seq footprint data (combined.fps.gz file) were downloaded from the European Bioinformatics Institute and any segments that were contained within a footprint region were positively classified and negatively otherwise. More technical detail about this data set can be found at the download link ftp://ftp.ebi.ac.uk/pub/databases/ensembl/encode/integration_data_jan2011/byDataType/footprints/jan2011/. Lastly, 690 ChIP-Seq data sets representing 161 unique regulatory factors, both generic and sequence specific, from 91 human cell types were downloaded from the Encode track at UCSC, at http://genome.ucsc.edu/cgi-bin/hgTrackUi?db=hg19&g=wgEncodeAwgTfbsUniform, using the union of all these data sets as genetic coordinates. Any segments completely contained within a region identified these tracks were positively classified and negatively otherwise. These classification rules were applied in the same way to segments in each cluster and the outcomes used to estimate the performance of each classifier and to construct a composite classifier indicative of the type of element found in each cluster.

#### Estimating binary classifier performance

The performance of a classifier is quantified in terms of sensitivity and specificity. These measure the proportions of true positive and true negative individuals (respectively) that are correctly classified. They are also known as the true positive and true negative rates, respectively. Ideally, these rates can be estimated using a “gold standard” benchmark data set for which the true classification of each individual is known.

When benchmark data is unavailable, alternative means must be used to estimate these quantities. We approach this by implementing a hierarchical Bayesian model detailed in Keith et al. [61]. In this model, the observed outcomes of each classifier for each segment are modelled as independent Bernoulli trials when conditioning on the unobserved true classification of each segment. The segments under consideration are assumed to come from a random sample of a wider population where the feature of interest is present in some proportion. Prior distributions are also assigned for the sensitivities and specificities of each of the classifiers and the hyper-parameters for the Bernoulli distributions in the model. Combined with the likelihood function obtained from the observed classifications, Bayes’ theorem can be employed to estimate the parameters of interest. Additional detail regarding the model and method is available in Appendix B.

#### Combining classifiers

Combinations of classifiers based on the union, intersection and negation operations are systematically enumerated, as described in [61]. Under the assumption of conditional independence between classifiers, the performance of these logical combinations can be computed easily with arithmetic arising from the definitions of sensitivity and specificity. Description of these arithmetic formulae can be found in Appendix C. The criteria used to select the best combination should reflect the relative importance assigned to sensitivity and specificity. We are interested in an optimal combination of classifiers, in the sense that this combination ranks highest in multiple criteria in a probabilistic sense:

- the product of sensitivity and specificity,
- the sum of absolute value of sensitivity and specificity,
- the sum of squares of sensitivity and specificity,
- the minimum of sensitivity and specificity.

Each criteria can present a different combination of classifiers as optimal. A consensus method for determining the best combination of classifiers is to select the combination that is optimal according to the greatest number of the four criteria; ties were not observed.

### Biological analysis

#### Ontology analysis

Clusters of segments were uploaded to the GREAT [20] platform for ontology association using the standard association rules. Genes were said to be associated with submitted regions if the region falls in a window 5kb downstream and 10kb upstream of the transcription start site of the gene, plus a distal extension of 1Mb in each direction. This is defined to be the regulatory domain of a gene. Ontology term enrichments are then computed with a binomial test over the set of the submitted genetic regions and gene annotations and only results achieving the significance threshold of 0.05 false discovery rate are reported.

**Fig. 7:**
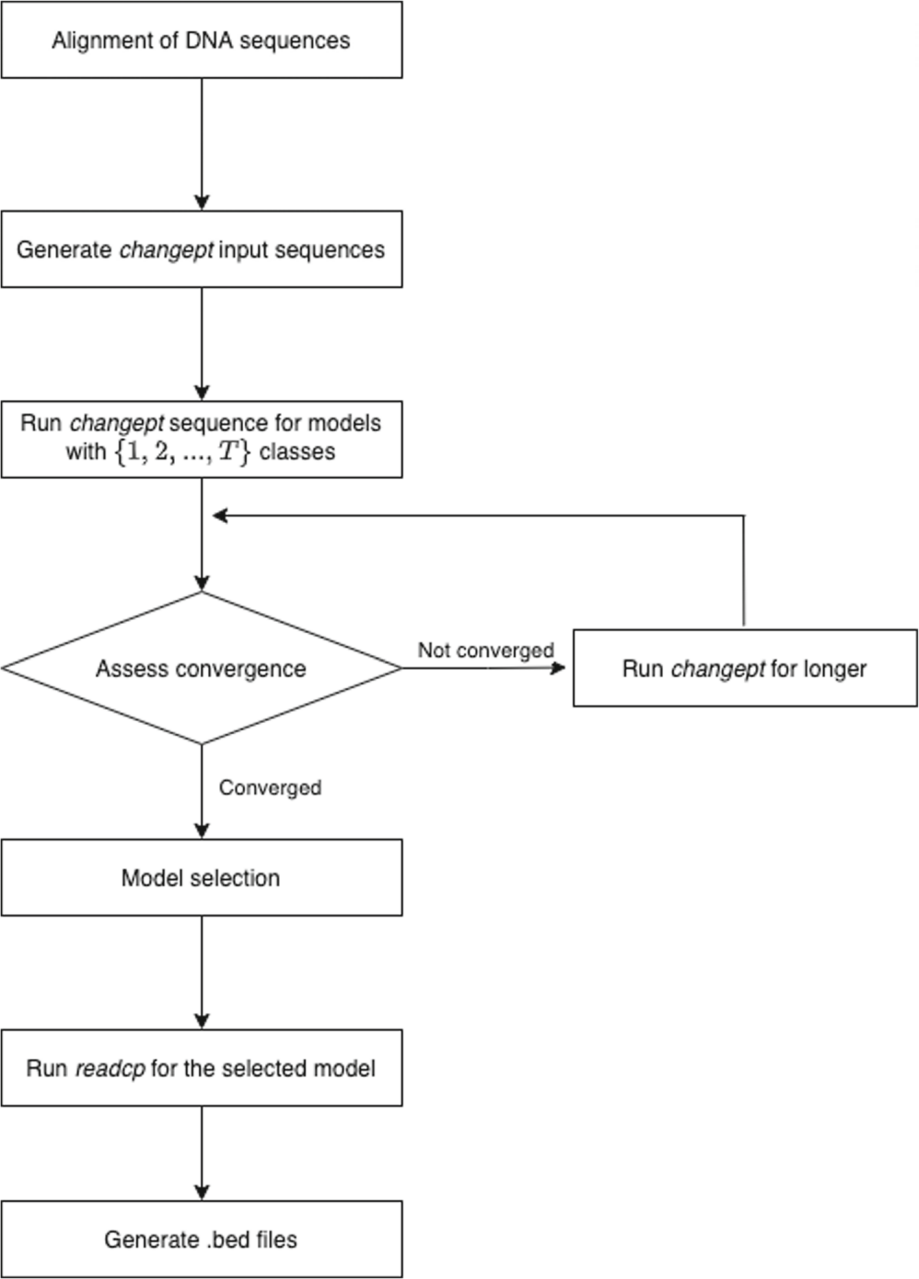
Flow diagram for changept analysis. The above figure is a reproduction from Algama [56] and summarises the steps involved in the segmentation classification portion of this analysis that is performed by changept

#### Motif analysis

Finally, to identify motifs, each cluster was uploaded to Trawler in duplicates using the standard parameters. Trawler identifies any motifs within the uploaded regions and tests them for significance against a random set of genetic regions to establish significance [62–64]. For each cluster, we then investigated the top three most statistically significant results to identify common motifs or cores. These motifs are presented alongside any positive matches found by Trawler in a known TFBS database and full.

### Computing requirements

Scripts were written in python and shell, while changept was written in C++, with additional analysis being performed in R. Each iteration of changept is linear in sequence length, but how the number of iterations required scales is unknown. In practice, we find that the iteration scale sublinearly in sequence length.

## Appendices

### Appendix A - flow diagram of changept analysis

This appendix contains a flow diagram for model selection process involved in changept.

### Appendix B - estimation of sensitivity and specificity in the absence of a gold standard

Suppose that there are *N* individuals, with *K* binary classifiers applied to each individual. Further, let *C*_*kn*_=1 denote classifier *k* producing a positive classification for individual *n*, similarly *C*_*kn*_=0 for a negative classification. The outcomes of these tests are modelled as independent trials, conditional on the true classification of individual *n*, *T*_*n*_. That is, the outcomes of the binary classifiers are conditinally independent, given *T*_*n*_. The equation

$$ P(C_{kn}|T_{n},\alpha_{k},\beta_{k}) = \left\{\begin{array}{ll} \alpha_{k}, & C_{kn}=1,T_{n}=1\\ 1-\alpha_{k}, & C_{kn}=0,T_{n}=1\\ \beta_{k}, & C_{kn}=1,T_{n}=0\\ 1-\beta_{k}, & C_{kn}=0,T_{n}=0 \end{array}\right. $$

describes the probability of a classification conditional on the underlying true class membership of an individual where *α*_*k*_ and *β*_*k*_ denote the true and false positive rates of the classifier.

A hierarchical Bayesian model is constructed in order to estimate the sensitivity and specificity of each of the *K* classifiers, which are *α*_*k*_ and 1−*β*_*k*_ respectively. We assume *ϕ* represents the proportion of the population that is condition positive for the feature of interest. The *N* individuals selected uniformly without replacement form a representative sample of the population, which allows us to model *T*_*n*_ for the *N* individuals as independent Bernoulli trials with parameter *ϕ*. Finally, priors are assigned to the parameters *ϕ*,*α*_*k*_ and *β*_*k*_. As in the original paper, we adopted uniform priors for these parameters. Additionally, the restriction that *α*_*k*_≥*β*_*k*_ is also followed.

### Appendix C - combining binary classifiers

The assumption of conditional independence also allows calculation of the sensitivity and specificity of logical combinations of binary classifiers. These logical combinations are constructed with the logical operators AND (∧), OR (∨) and NOT (¬). In the following, the variables *X* and *Y* are used to denote both classifiers and the set of individuals classified as positive for that classifier. The sensitivity of an intersection is given by:

$$\begin{array}{*{20}l} \text{SENS}(X \wedge T) &= P(X \cap Y | T)\\ &= P(X|T)\times P(Y|T)\\ & = \text{SENS}(X)\times \text{SENS}(Y) \end{array} $$

with specificity

$$\begin{aligned} \text{SPEC}(X \wedge T) &= P((X\cap Y)^{c}|T^{c})\\ &= P(X^{c} \cup Y^{c}|T^{c})\\ &= P(X^{c}|T^{c}) + P(Y^{c}|T^{c}) - P(X^{c}|T^{c})\times P(Y^{c}|T^{c}) \\ &= \text{SPEC}(X) + \text{SPEC}(Y) - \text{SPEC}(X)\times\text{SPEC}(Y). \end{aligned} $$

Using intersections, the space of possible combinations can be partitioned into 2^*K*^ disjoint regions. Then, using unions of these disjoint regions, the following formulae can be used to compute sensitivity:

$$\begin{array}{*{20}l} \text{SENS}(X \vee Y) &= P(X\cup Y|T)\\ &= P(X|T) + P(Y|T)\\ &= \text{SENS}(X) + \text{SENS}(Y) \end{array} $$

and specificity:

$$\begin{array}{*{20}l} \text{SPEC}(X \vee Y) &= P((X \cup Y)^{c}|T^{c})\\ &= P(X^{c}\cap Y^{c} | T^{c})\\ &= P(X^{c}| T^{c}) + P(Y^{c} | T^{c}) - 1\\ &= \text{SPEC}(X) + \text{SPEC}(Y) - 1. \end{array} $$

The above two formula are sufficient to systematically evaluate all $2^{2^{K}}$ possible combinations of binary classifier outcomes.

## Supplementary Information

**Additional file 1** ROC curves for GC content

## Data Availability

The dataset(s) supporting the conclusions of this article are available in the maderazo2020detection repository, https://melbourne.figshare.com/projects/maderazo2020detection/81830, with the repository being organised into folders. The multiz8way.maf.gz file containing the 8 way zebrafish reference alignment was downloaded directly from UCSC at the following URL: https://hgdownload-test.gi.ucsc.edu/goldenPath/danRer7/multiz8way/. DNAse-Seq data was downloaded directly from this URL: ftp://ftp.ebi.ac.uk/pub/databases/ensembl/encode/integration_data_jan2011/byDataType/footprints/jan2011/ and TFBS datat was taken from the following UCSC URL: http://genome.ucsc.edu/cgi-bin/hgTrackUi?db=hg19&g=wgEncodeAwgTfbsUniform. The necessary code to compile and run changept to perfom segmentation including readcp for segment retrieval is available at the following github repository https://github.com/dmaderazo/maderazo2020detection. Scripts for generating trace plots of the log likelihood to establish burn in and plot information critetion and mixture proportions are also available at the repository.

## Abbreviations

CRE: Cis-regulatory elements
CRM: Cis-regulatory module
ChIP-Seq: Chromatin immuno-precipitation followed by sequencing
CSRP3: Cysteine and glycine rich protein 3
DHS: DNase hypersensitivity
DNA: Deoxyriboucleic acid
ERC2: ELKS/RAB6-interacting/CAST family member 2
FLRT: Fibronectin leucine rich transmembrane
FOX: Forkhead box
GC: Guanine-cytosine
GO: Gene ontology
GREAT: Genomic regions enrichment of annotations tool
PAX: Paired box
PFE: Putative functional element
POU: Pit, oct, and unc
RNA: Ribonucleic acid
TF: Transcription factor
TFBS: Transcription factor binding site
TSS: Transcription start site
UCSC: University California Santa Cruz
ZNF: Zinc finger
