# Supplementary material for: Detection and identification of cis-regulatory elements using change-point and classification algorithms
Source: BMC Genomics. 2022 Jan 25;23:78. doi: 10.1186/s12864-021-08190-0 (PMC8790847; doi:10.1186/s12864-021-08190-0)
Supplement: Supplementary file 1 — Additional file 1 ROC curves for GC content [file 12864_2021_8190_MOESM1_ESM.pdf]

Supplementary File 1: ROC Curves for GC Content

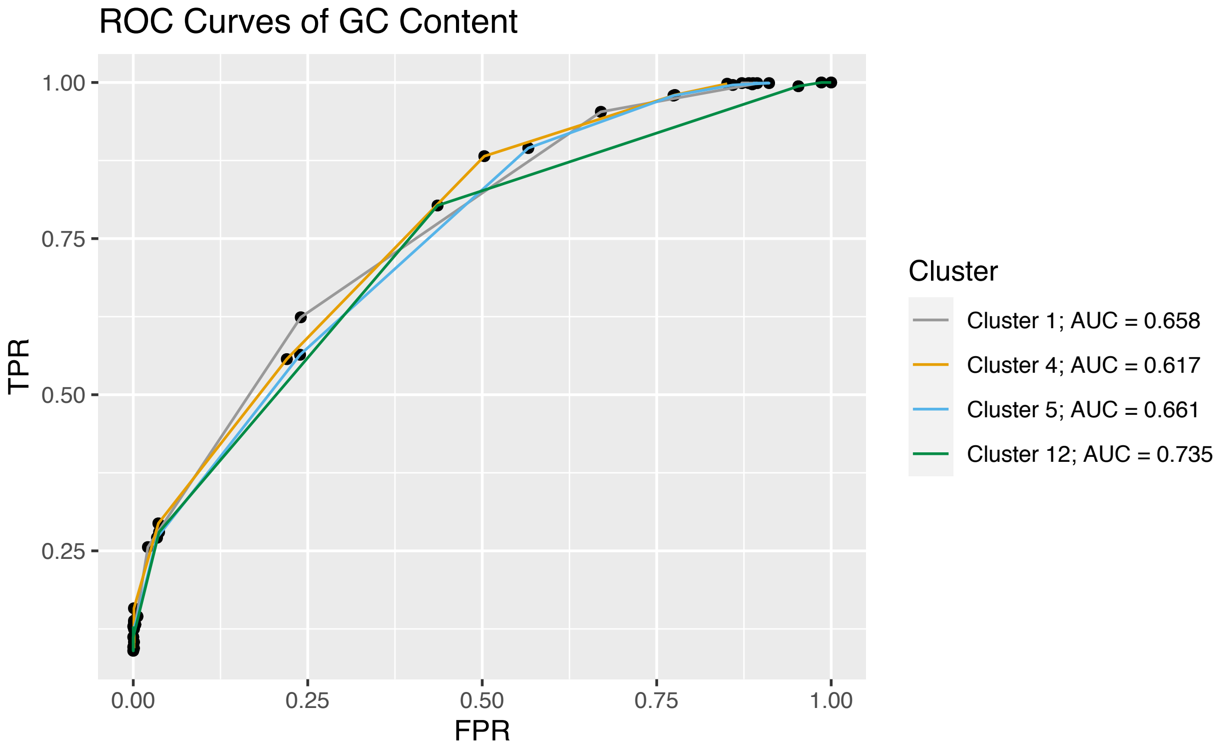

Supplementary Figure 1: **ROC curves.** By varying the threshold value for GC content for the clusters presented in the results of the paper. In each of the clusters, the AUC values are given above.
